# Supplementary material for: Pathophysiological Significance of Neutrophilic Transfer RNA-Derived Small RNAs in Asymptomatic Moyamoya Disease
Source: Cells. 2021 May 1;10(5):1086. doi: 10.3390/cells10051086 (PMC8147334; doi:10.3390/cells10051086)

2D Structure	Local AU	Position

```
128                                     159
                                 8mer-1a
 5'-catgggCTTCACAGCATGGCACACATGTGGGa-3' utr
        |:| ||  || |:| || |||||||
 3'-uccuuaGGAUUGGCG-AUC-UG-GUACACCCu-5' tRF
                1615 1413     7 6 5 4 3 2
             3' Pairing        Seed
```

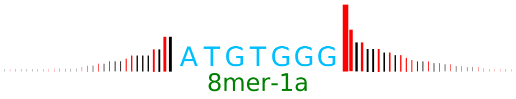

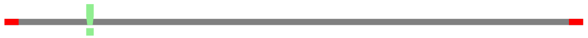

2D Structure	Local AU	Position

```
79                                    106
                                8mer-1a
 5'-gtctgtgtgtACTGTTA-AGTGTGTGGGa-3' utr
          ||:|:||  |  ::||||||
 3'-uccuuaggauUGGCGAUCUGGUACACCCu-5' tRF
               16151413     7 6 5 4 3 2
             3' Pairing       Seed
```

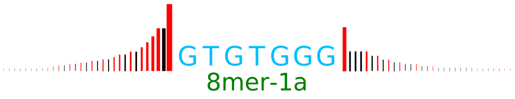

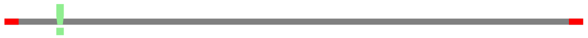

Supplement: Supplementary file 1 [file cells-10-01086-s001.zip › cells-1149161 3rd supplementary materials/Data Supplements/Data Supplement V.zip!/Data supplement 6-mRNAs targets of tsRNAs/tsRNA-Glu-TTC-010/Diagrams/tRF-Glu-TTC-010_vs_ENST00000418425(LETMD1).pdf]
